# Impact of paravertebral blocks on analgesic and non-analgesic outcomes after video-assisted thoracoscopic surgery: A propensity matched cohort study

**DOI:** 10.1371/journal.pone.0252059

**Published:** 2021-05-20

**Authors:** Yatish S. Ranganath, Vendhan Ramanujam, Yoshiko Onodera, John Keech, Evgeny Arshava, Kalpaj R. Parekh, Rakesh V. Sondekoppam

**Affiliations:** 1 Department of Anesthesia, University of Iowa Carver College of Medicine, Iowa City, Iowa, United States of America; 2 Department of Anesthesiology, Warren Alpert Medical School of Brown University, Providence, Rhode Island, United States of America; 3 Department of Anesthesiology and Critical Care Medicine, Asahikawa Medical University, Asahikawa, Japan; 4 Department of Surgery – Cardiothoracic Surgery, University of Iowa Carver College of Medicine, Iowa City, Iowa, United States of America; Ohio State University Wexner Medical Center Department of Surgery, UNITED STATES

## Abstract

**Background:**

Regional analgesic techniques such as paravertebral blocks (PVBs) have been popularized for analgesia following video-assisted thoracoscopic surgery (VATS). In this single center retrospective propensity matched cohort of subjects, we investigate the impact of paravertebral blocks on the analgesic and non-analgesic outcomes.

**Methods:**

Institutional database was queried to identify all patients undergoing VATS between January 2013 and July 2019 and these patients were divided into those who received paravertebral blocks in combination with general anesthesia (GA) [PVB group] and those who received GA without paravertebral blocks [GA group]. Propensity score matching based on common patient confounders were used to identify patients in each group. Primary outcomes of the study were average pain scores and opioid consumption in the first 24 hours. Secondary analgesic outcomes included pain scores and opioid requirements at other timepoints over the first 48 hours. Non analgesic outcomes were obtained from STS General Thoracic Surgery Database and included length of hospital stay, need for ICU admission, composite outcome of any complication during the hospital course and 30-day mortality. Exploratory analyses were conducted to investigate the impact of PVB on analgesia following different types of surgery and as to whether any other covariates had a greater influence on the included patient centered outcomes.

**Main results:**

After propensity score matching, a total of 520 patients (260 per group) were selected for the study out of 1095 patients. The opioid consumption in terms of oral morphine milligram equivalent (MME) [Median (IQR)] for the first 24 hours was significantly lower with the use of PVB [PVB group– 78.5 (96.75); GA group—127.0 (111.5); p<0.001] while the average pain scores in the first 24 hours did not differ significantly [PVB group—4.71 (2.28); GA group—4.85 (2.30); p = 0.70]. The length of hospital stay, opioid requirements at other timepoints, need for ICU admission in the immediate post-operative period and the composite outcome–‘any complication’ (35% vs 48%) were significantly lower with the use of PVB. Subgroup analysis showed a longer duration of benefit following major lung surgeries compared to others.

**Conclusion:**

Paravertebral blocks reduced the length of stay and opioid consumption up to 48 hours after VATS without significantly impacting pain scores.

## Introduction

Video-assisted thoracoscopic surgery (VATS) has reshaped the field of thoracic surgery by greatly decreasing the need for open thoracotomy. Several societies have published guidelines recommending VATS as the operative approach of choice for early stage lung cancer due to better postoperative outcomes and less surgical trauma with VATS approach compared to open surgery [1–3] and these findings have also been reconfirmed in two recent randomized trials [4–6]. Despite these advantages, VATS approaches still result in a significant number of patients having moderate to severe postoperative pain and, approximately 25%–47% of patients end up suffering from persistent postsurgical pain similar to that seen after open thoracotomies [7–12]. One of the predictors to the development of chronic pain is a poorly controlled postsurgical pain [8] and hence it is imperative to look for effective analgesic modalities in the context of VATS surgery to reduce both immediate- and long-term morbidity in these patients.

The ideal regimen for post-VATS analgesia is not clear, but a multimodal analgesic regimen utilizing regional analgesic technique seems promising. Several regional anesthesia techniques have been investigated [13–16], but a clear consensus regarding the ideal technique is lacking as shown in a recent systematic review [17]. Studies evaluating the effectiveness of paravertebral blocks for VATS patients, while having shown to provide acceptable analgesia [18–23], are often critiqued for their small sample size and the non-inclusion of other patient outcomes. Hence, evidence from a larger sample of patient population undergoing a variety of VATS surgeries is warranted and the current study is one such attempt to evaluate the impact of PVB on analgesic and patient outcomes.

The primary aim of our study was to compare analgesia as assessed by a composite outcome of pain scores and opioid consumption in the first 24 hours between 2 groups: patients undergoing VATS with paravertebral blocks in combination with GA (PVB group) and; patients undergoing VATS with GA without paravertebral blocks (GA group). Our secondary aim was to examine the difference in patient outcome measures between the two groups by analyzing the length of stay and the other perioperative data collected from the Society of Thoracic Surgery (STS) Database.

## Methods

With the approval of the University of Iowa Institutional Review Board (HAWK IRB ID #201903886- October 2019), we obtained data on adult patients undergoing VATS between January 2013 and July 2019 at the University of Iowa Hospitals and Clinics. Requirement for written informed consent was waived by Institutional Review Board for this retrospective study.

### Study population

All adult patients aged 18 years or older who had VAT surgeries at the University of Iowa Hospitals and Clinics were included. Our exclusion criteria included: Patients younger than 18 years; use of regional analgesic techniques other than PVB; ASA physical status V and above; primary thoracotomy or conversion of VATS to open thoracotomy; esophagectomies; missing data for the baseline characteristics. Patients who declined research authorization were excluded. The reviewed VATS cases were divided into two groups: VATS cases with paravertebral blocks in combination with GA (PVB group) and VATS cases with GA without paravertebral blocks (GA group). It is our institutional practice to perform single injection paravertebral blocks between T4-T7 interspaces using 20 to 30 ml of 0.5% Ropivacaine.

### Database and study setting

Data were collected from the patient’s electronic medical record database used by our institution (Epic systems software, Verona, WI, USA) and the Society of Thoracic Surgery (STS) database. The primary STS database was matched and merged with EPIC database containing pain scores and opioid medications using patient identification number and their date of surgery. Analgesic outcomes (pain scores and opioid consumption) were obtained from our electronic medical record database. All other outcomes including patient comorbidities and non-analgesic outcomes were extracted from the STS database. The EPIC data and the STS database data were combined by matching patient identifiers and date of surgery. Subsequently, patients not meeting the inclusion criteria were removed from the combined database and then all patient information was anonymized and de-identified prior to analysis.

Analgesic outcome data were collected by our perioperative and floor nursing as a standard of care. Post-anesthesia care unit and floor nurses assessed and recorded 10-point numeric rating scale (NRS) pain scores every 4 hours after discharge from PACU throughout their hospital stay duration. Data on pain scores and postoperative opioid consumption was obtained from electronic medical records which was converted into oral MME doses using the opioid conversion charts [24].

The STS General Thoracic Surgery Database (GTSD) is the largest clinical thoracic surgical database in North America currently having more than 1,000 participating surgeons. The STS database has 4 components (adult cardiac surgery; general thoracic surgery; congenital cardiac surgery and intermacs—a registry for the clinical outcomes of patients who receive an FDA-approved mechanical circulatory support device to treat advanced heart failure in North America), each focusing on a different area of cardiothoracic surgery and has been used in our institution since 2006.

Covariates important to the pain and postoperative outcomes were identified prior to the data extraction. These included age, sex, BMI, smoking status, pre-operative chemo or radiotherapy, pre-operative use of opioids, ASA physical status class (1, 2, 3, 4), type of surgery, duration of surgery and presence of comorbidities. The type of surgery was classified into major lung surgery, minor lung surgery and non-lung surgery and the full list of surgery types and their classification is available in S1 Table. The comorbidities included as covariates for matching comprised hypertension, diabetes, COPD, CHF, peripheral vascular disease, coronary artery disease, history of lung cancer.

### Outcome measures

Our primary outcomes were time-weighted average NRS pain scores and total postoperative oral MME dose of opioids evaluated over the first 24 postoperative hours. Secondary outcome measures included NRS pain scores and oral MME consumption between 24 to 48 hours, length of stay, need for ICU admission, composite outcome of ‘any complications’ and 30-day mortality. Composite outcome of ‘any complications’ in the STS database is listed as “postoperative events occurred” and indicates if the patient suffered from any complications during their hospital course. A complete list of complications recorded under this heading in the STS database is provided in S2 Table.

### Statistical analysis

Patients in the PVB group and the GA group were matched using propensity scoring in a 1:1 ratio. Propensity scores (estimated probability of receiving paravertebral block) was determined using the PS match plugin for R in SPSS (IBM Corp. Released 2017. IBM SPSS Statistics for Windows, Version 25.0. Armonk, NY: IBM Corp.) and the optimal match function was utilized with a caliper of 0.2. All covariates were included in the model without further correction. We did not include the year of surgery as a covariate but analyzed the impact of the year of surgery exploratorily. Balance of covariates was assessed, and a significant imbalance of covariates was said to be present if the standardized mean difference (SMD) was more than 10% between the two groups [25, 26]. Data distribution was analyzed using quantile-quantile plot followed by Shapiro-Wilk test. Continuous data is presented as Mean (± SD) or Median (IQR) depending on the distribution, while the ordinal data is presented as frequencies. Baseline demographics and distribution of covariates were crosschecked for matching using inter group comparison and the summary data of baseline patient characteristics is provided in Table 1. The groups were then compared using Mann-Whitney U-test for continuous outcomes while dichotomous outcomes such as need for ICU stay or presence of any post-operative complications were analyzed using chi-square test. Any secondary outcomes with missing data were not analyzed further.

**Table 1:** Patient characteristics and surgical factors.

Variable	GA (n = 260)	PVB (n = 260)	MD (95% CI)	P-value (sig < 0.05)
	Mean (± SD)	Mean (± SD)		
Age	60.62 (± 15.43)	60.35 (± 15.24)	0.26 (-2.37; 2.90)	0.84
Sex (F:M)	121:139	126:134		0.66
BMI	28.18 (± 6.64)	28.17 (± 6.22)	0.01 (-1.09; 1.11)	0.98
ASA class (1:2:3:4)	4:102: 146: 8	7: 94:152:7		0.72
Surgical class				0.72
• Non-lung surgery	14	11		
• Minor lung surgery	147	143		
• Major lung surgery	99	106		
Duration of surgery (hr)	2:01 (± 1.12)	2:06 (± 1.57)	0:08 (-0:22; 0.11)	0.52

Percentage of patients requiring less than 50 MME in the first 24 hours, less than 100 MME in the first 48 postoperative hours and those requiring less than 3 days of length of stay are summarized by cross tabulation. To know the influence of covariates on 24- and 48-hour morphine consumption (dichotomized to 50 and 100 mg respectively), binary logistic regression was performed using all relevant covariates. All statistical tests were conducted using SPSS version 25 (IBM Corp. Released 2017. IBM SPSS Statistics for Windows, Version 25.0. Armonk, NY: IBM Corp.) and p-value < 0.05 was considered to be statistically significant.

## Results

**Fig 1:**
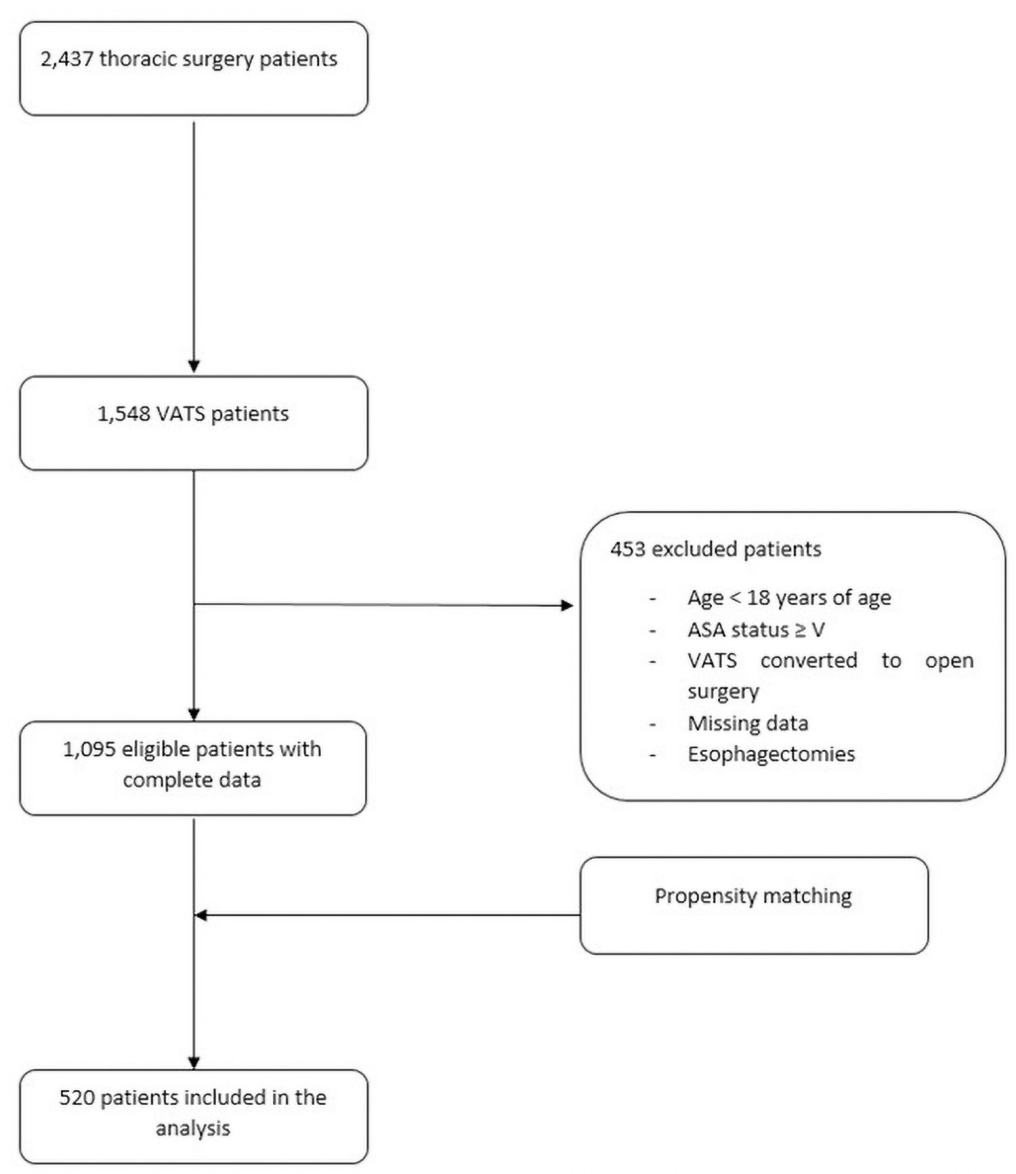
Flow chart of patient selection.

Our query of the electronic medical records revealed 1548 VAT surgeries and after eliminating 453 patients, 1095 patients remained. The patients meeting the eligibility criteria for the study included 294 patients who underwent VATS with paravertebral blocks in combination with general anesthesia (GA) (PVB group) and 801 patients who received GA without paravertebral blocks (GA group). Based on demographic and baseline characteristics, we successfully 1:1 matched a total of 520 patients with 260 patients per group (Fig 1). Tables 1 and 2 show the group characteristics after propensity score matching; all patients’ baseline characteristics and surgical factors no longer significantly differed among the two groups.

**Table 2:** Other covariates included for propensity score matching and their distribution in the study cohorts.

Variable	GA	PVB	P-value
Hypertension (N:Y)	113:147	116:144	0.79
Diabetes (N:Y)	220:40	228:32	0.31
Peripheral vascular disease (N:Y)	250:10	246:14	0.40
CHF (N:Y)	257:3	257:3	1.00
CAD (N:Y)	224:36	216:44	0.33
COPD (N:Y)	203:57	201:59	0.83
History of Lung Cancer (N:Y)	147:113	137:123	0.37
Prior CTS (N:Y)	232:28	231:29	0.88
Preoperative thoracic radiation therapy (N:Y)	258:2	256:4	0.41
Opioid (N:Y)	224:36	218:42	0.46
Smoking (N:Y)	116:144	103:157	0.23

**Fig 2:**
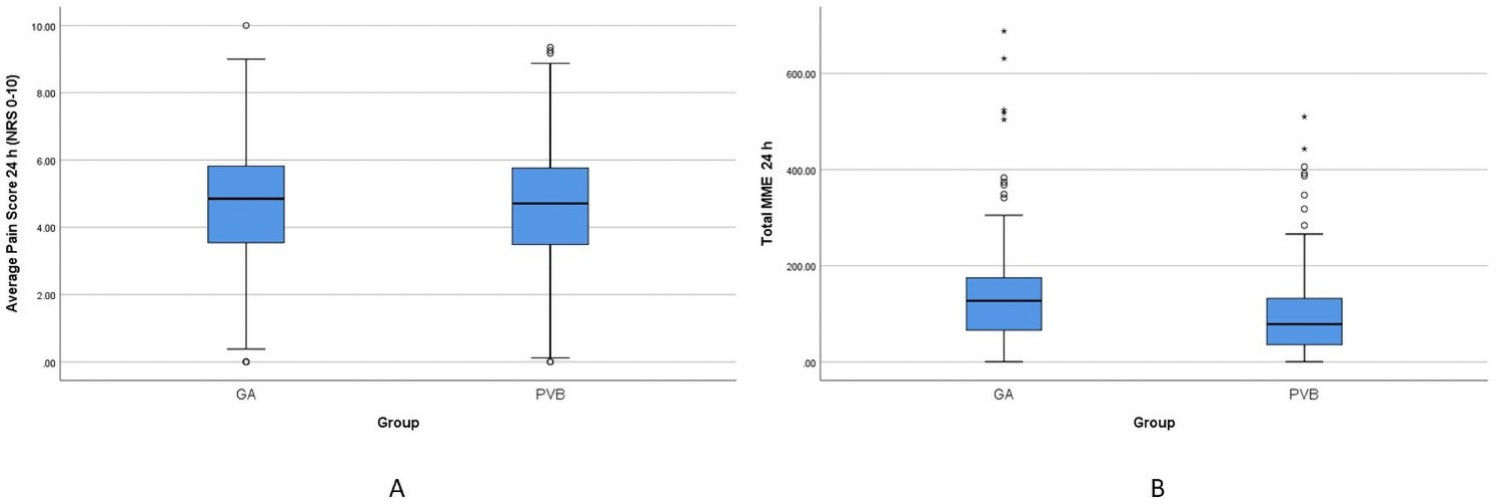
A. Average numeric rating scale (NRS) pain scores in the first 24 postoperative hours in the 2 groups. B. Opiod consumption in terms of oral morphine milligram equivalents (MME) at 24 hours in the 2 groups.

Table 3 shows the results of postoperative pain scores and opioid requirements. The average pain scores in the first 24 hours did not differ significantly between the 2 groups [median (IQR) PVB—4.71 (2.28); GA—4.85 (2.30); p = 0.70] (Fig 2). However, the opioid consumption in terms of oral MME for the first 24 hours was significantly lower in the PVB group compared to the GA group [median (IQR) PVB– 78.5 (96.75); GA-127.0 (111.5); p<0.001]. Oral MME was also significantly lower in the PVB group in the 24 to 48-hour period [median (IQR) GA– 48.0 (102.63); PVB– 32.0 (71.0); p = 0.007] but average pain scores during this period did not differ significantly [median (IQR) PVB– 3.59 (2.75); GA– 3.86 (2.67); p = 0.37]. Table 4 shows the results for the other secondary outcomes. The length of stay [GA– 4.0 (3.0); PVB– 3.0 (3.0) P = 0.002], need for ICU admission (GA– 14.2% vs PVB– 9.2%) and the composite outcome–any complication (GA– 45.8% vs PVB– 35%) were significantly lower in the PVB group. 30-day mortality was not different between the two groups.

**Table 3:** Postoperative pain scores and opioid requirements.

Variable	GA	PVB	P-value
	Median (IQR)	Median (IQR)	
Max pain score at 24 h	8.00 (3)	8.00 (3)	0.92
Avg pain score at 24 h	4.85 (2.30)	4.71 (2.28)	0.70
Max pain score at 24–48 h	7.00 (3.00)	6.00 (4.00)	0.09
Avg pain score at 24–48 h	3.86 (2.67)	3.59 (2.75)	0.37
MME 24 h	127.00 (111.50)	78.5 (96.75)	**<0.001**
MME 24–48 h	48.00 (102.63)	32.00 (71.00)	**0.007**
Total MME 0–48 h	158.00 (172.24)	116.00 (135.75)	**<0.001**

**Table 4:** Other secondary outcomes.

Variable	GA (n = 260)	PVB (n = 260)	P-value
Length of stay	4.0 (3.0)	3.0 (3.0)	**0.002**
Patient disposition			< 0.001
• ICU	29	14	
• Intermediate care unit	115	177	
• Outpatient/obs unit	10	12	
• Regular floor bed	106	57	
Need for ICU this entire admission (N:Y)	223:37	236:24	0.07
Mortality at 30 days (N:Y)	258:2	259:1	0.56
Any complication (N:Y)	141:119	169:91	0.012

Subgroup analysis based on the type of surgery found a significant benefit for 24-hour MME for all types of surgeries, but the benefit persisted at 24–48 hour MME in major lung surgeries only (S3 Table). There was also a significant difference in length of stay between the two groups following minor and major lung surgeries.

### Regression analysis on opioid consumption and length of stay

A logistic regression performed to ascertain the effects of confounders affecting postoperative opioid usage dichotomized the opioid use at a cutoff of less/more than 50 MME of opioids in the first 24 postoperative hours and for 100 MME of opioids at 48 postoperative hours. Factors considered in the model included paravertebral block, age, BMI, gender, type of surgery, history of chronic opioid usage, ASA class, presence of peripheral vascular disease, history of lung cancer, prior cardiothoracic surgery and prior thoracic radiation. The logistic regression model for the probability of using less than 50 MME of opioid in the first 24 hours was statistically significant, χ2(11) = 59.08, p < .0001. The model explained 16.10% (Nagelkerke R2) of the variance in opioid consumption for the likelihood of using less than 50 MME at 24 hours and correctly classified 77.5% of cases. Odds of using less than 50 MME in the first 24 postoperative hours was 2.69 times more likely with the use of PVB than in GA group (Table 5). Increasing age and ASA class were also associated with an increased likelihood of using less than 50 MME in the first 24 hours. A similar association was seen for the logistic regression model for the dichotomized outcome of using less than 100 MME in the first 48 postoperative hours, χ2(20) = 93.20, p < .0001 (Nagelkerke R2 = 22.5% and the model predictive percentage = 70.3%) with use of PVB showing the greatest effect (odds ratio = 2.43, p<0.001). Other covariates affecting the model to a lesser degree included age, history of COPD, history of hypertension and ASA class.

**Table 5:** Impact of PVB on opioid consumption and length of stay.

Variable	GA (%)	PVB (%)	Odds ratio	P-value
MME< 50 mg in 24 h	41 (15.76%)	82 (31.53%)	2.69	<0.001
MME< 100 mg in 48 h	74 (28.46%)	116 (44.61%)	2.65	<0.001
Stay < 3 days	113 (43.46%)	143 (55%)	1.80	0.002

Similarly, a logistic regression performed to ascertain the effects of factors important for the length of stay dichotomized the duration of hospital stay to less/more than 3 days. Factors considered in the model included PVB, age, BMI, PVD, history of lung cancer, prior CTS, preoperative thoracic radiation therapy, chronic opioid usage, gender, ASA class, type of surgery, hypertension, diabetes, CHF, CAD, COPD, smoking status. The logistic regression model for the probability of staying less than 3 days following surgery was statistically significant, χ2(17) = 85.15, p < .0001. The model explained 20.2% (Nagelkerke R2) of the variance for the likelihood of staying less than 3 days postoperatively and correctly classified 68.0% of cases. Odds of staying less than 3 postoperative days with the use of PVB was 1.80 times more likely to that in GA group. Increasing ASA class (OR = 1.62; p = 0.009), type of surgery (OR = 2.35; p = 0.001) and presence of COPD (0.58; p = 0.034) had a significant influence on the model more than from the use of PVB. A similar logistic regression model looking into the association of the same covariates on the incidence of “any complications” during the hospital course was statistically significant χ2(21) = 62.86, p < .0001 and the model explained 15.4% (Nagelkerke R2) of variance and correctly classified 65.5% of the cases. Among all the covariates, type of surgery (minor vs major) (OR: 1.04; p = 0.006) and gender (F:M) (OR: 0.58; p = 0.007) showed a better association than the use of paravertebral block (OR: 0.52; p = 0.003).

## Discussion

In this retrospective review of over 1,000 patients undergoing VAT surgery, we identified several significant associations. Use of paravertebral block was associated with a reduction in opioid consumption at 24 hours and 48 hours following VATS but there was no significant benefit in terms of average or maximum pain scores at any timepoints. The association of opioid sparing with the use of PVB was present for all types of surgeries at the 24-hour timepoint, but this benefit persisted at 24–48 hours following major lung surgeries only. Patients receiving PVBs were also more likely to require < 3 days of length of stay.

Acute pain after VATS is often complex and multifactorial, attributed to the following factors: *nociceptive pain* caused by surgical trauma to muscular and bony structures of the thorax; *neuropathic pain* due to intercostal nerve irritation and; *referred pain* [27] transmitted by the phrenic nerve following irritation of the pleura or pericardium, which leads to ipsilateral shoulder pain. While we did not find any difference in pain scores between the groups, this could partly be due to the multiple sources of pain, all of which may not be covered with the use of paravertebral block or due to the fact that the timepoints of the study are well past the duration of the block and hence, strategies to prolong the duration of analgesia sufficient to cover the duration of significant pain are essential.

A major finding of our study was lower opioid consumption over the first 48 hours in the PVB group. While it is uncertain as to whether the use of nerve blockade impacts long term opioid consumption, opioid overprescribing after thoracic surgery is fairly common [28–30]. Increased in-hospital use of opioids and a decreased opioid free interval before discharge are known to be associated with increased post-discharge opioid usage and long-term opioid use [31]. Hence, advocating for a regional anesthesia based multimodal analgesia regimens may embrace the promise of minimizing in-hospital opioid usage and its subsequent benefits.

Studies evaluating the use of continuous regional analgesia techniques for VATS are limited. Unlike open thoracotomy where PVBs have been shown to provide analgesia similar to thoracic epidural analgesia (TEA), VATS studies comparing PVBs with TEA need better level of evidence due to the low number of studies with equivocal evidence [32–35]. Among the several studies evaluating the utility of PVBs for VATS over the last 2 decades, Vogt et al. showed significant improvement in pain scores that persisted for 48 h postoperatively but there was no difference in morphine consumption at 24 h or 48 h [23]. In contrast, Hill et al. demonstrated significant reduction in opioid consumption and pain scores 6 h after surgery; however, a longer lasting effect was not seen [19]. Kaya et al. reported lower pain scores at 1, 2, and 4 h after surgery but similar to our study findings, there were no significant differences in pain scores at later timepoints while the cumulative morphine requirements were significantly lower in the PVB group throughout the 48-h study period (except at the 12 h datum point) [21]. Unlike the study by Kaya et al, we did not collect the pain scores between the 2 groups in the first few hours after surgery and hence our study cannot comment on the early analgesic benefit of single injection PVB. While earlier studies mainly focus on analgesics outcomes, our study evaluates non-analgesic outcomes from the STS database in addition to the regular analgesic outcomes, adding to the growing body of literature demonstrating the beneficial impact of PVBs.

The strengths of our study, apart from a larger sample of patients, is the inclusion of data from the STS database that enabled us to examine the association between the use of PVBs with non-analgesic outcomes important to VATS surgery. As with other retrospective studies, our ability to adjust for potential confounding is limited to available data. Although we accounted for confounding effects of seventeen patient and surgical factors, residual bias due to uncontrolled confounding variables remains possible. Potential residual bias limits our ability to make causal conclusions and we can only deduce association in this present study. Our study findings need to be reconfirmed with well powered randomized trials.

In conclusion, our retrospective study showed that use of pre-operative single injection paravertebral blocks was associated with lower opioid requirement following VAT surgeries. Use of PVB was also associated with favorable non-analgesic benefits in terms of length of stay, need for ICU admission and the composite outcome for ‘any complications’.

## Supporting information

S1 Table Classification of type of lung surgery. (DOCX)

S2 Table Composite outcome for any complication. (DOCX)

S3 Table Subgroup analysis (based on type of surgery). (DOCX)

S1 Checklist STROBE statement—Checklist of items that should be included in reports of observational (cohort) studies. (DOCX)
