# Supplementary material for: Impact of paravertebral blocks on analgesic and non-analgesic outcomes after video-assisted thoracoscopic surgery: A propensity matched cohort study
Source: PLoS One. 2021 May 20;16(5):e0252059. doi: 10.1371/journal.pone.0252059 (PMC8136840; doi:10.1371/journal.pone.0252059)
Supplement: S1 Table — (DOCX) [file pone.0252059.s001.docx]

| **S1 Table: Classification of type of lung surgery.** |
| --- |
| **Minor lung surgery**  Thoracoscopy, diagnostic with biopsy of pleura  Thoracoscopy, diagnostic with biopsy of lung infiltrate  Thoracoscopy, diagnostic with biopsy of lung nodule or mass  Thoracoscopy with therapeutic wedge resection  Thoracoscopy with pleurodesis  Thoracoscopy with decortication  Thoracoscopy insertion of tunneled pleural catheter  Thoracoscopy with control of traumatic hemorrhage  Thoracoscopy with empyema evacuation  Thoracoscopy with pulmonary decortication  Thoracoscopy with removal of intrapleural foreign body or fibrin deposit  Thoracoscopy, diagnostic lungs and pleural space without biopsy  Thoracoscopy with excision-plication of bullae  Thoracoscopy with parietal pleurectomy  Thoracoscopy with partial pulmonary decortication  Thoracoscopy with total pulmonary decortication  **Major lung surgery**  Thoracoscopy with lobectomy  Thoracoscopy with pneumonectomy  Thoracoscopy with removal of lung  Thoracoscopy with segmentectomy  Thoracoscopy with total pneumonectomy  Thoracoscopy, diagnostic with diagnostic wedge resection followed by anatomic lung resection  **Non-lung surgery**  Thoracoscopy with excision of chest wall tumor  Thoracoscopy with excision of mediastinal cyst, tumor or mass  Thoracoscopy with excision of mediastinal cyst, tumor, or mass  Thoracoscopy with excision of pericardial cyst, tumor or mass  Thoracoscopy with lymphadenectomy  Thoracoscopy with partial resection of pericardial sac  Thoracoscopy with pericardial window  Thoracoscopy with pericardiostomy  Thoracoscopy with imbrication of diaphragm  Thoracoscopy with Suture of esophageal wound or injury  Thoracoscopy with thoracic sympathectomy  Thoracoscopy with thymectomy  Thoracoscopy with diaphragm repair  Ligation thoracic duct (38381)  Mediastinoscopy, with or without biopsy  Thoracoscopy with posterior mediastinal mass resection  Thoracoscopy, diagnostic pericardial sac, with biopsy  Thoracoscopy, diagnostic with biopsy of mediastinal space |
