# Supplementary material for: Impact of paravertebral blocks on analgesic and non-analgesic outcomes after video-assisted thoracoscopic surgery: A propensity matched cohort study
Source: PLoS One. 2021 May 20;16(5):e0252059. doi: 10.1371/journal.pone.0252059 (PMC8136840; doi:10.1371/journal.pone.0252059)
Supplement: S2 Table — (DOCX) [file pone.0252059.s002.docx]

| **S2 Table: Composite outcome for any complication** |
| --- |
| **Pulmonary:** Air leak > 5 days duration; Atelectasis requiring bronchoscopy; Pleural Effusion requiring drainage; Pneumonia; Acute Respiratory Distress Syndrome (ARDS); Respiratory Failure; Bronchopleural Fistula; Pulmonary Embolus; Pneumothorax requiring chest tube insertion; Initial Vent Support > 48 hours; Tracheostomy; Other pulmonary event  **Cardiovascular:** Atrial arrhythmia requiring treatment; Ventricular arrhythmia requiring treatment; Myocardial infarction; Deep venous thrombosis requiring treatment; Other cardiovascular event  **Gastrointestinal:** Ileus; Anastomotic leak requiring medical treatment only; Dilation esophagus; Conduit Necrosis requiring Surgery; Delayed conduit emptying requiring intervention (pyloric dilatation or botox) or maintenance of NG drainage > 7days; Clostridium Difficile infection; Other gastrointestinal event  **Hematology:** Packed red blood cells transfusion outside operating room (OR)  **Urologic:** Urinary tract infection; Urinary retention requiring catheterization; Discharged with Foley catheter  **Infection:** Empyema requiring treatment; Surgical Site Infection; Sepsis; Other Infection    **Neurology:** New central neurological event; Recurrent laryngeal nerve paresis -unexpected; Delirium; Other neurological event  **Miscellaneous:** New renal failure; Chylothorax requiring medical intervention; Other events requiring OR with general anesthesia; Postoperative invasive procedure; Unexpected Admission to ICU |
