# Supplementary material for: Impact of paravertebral blocks on analgesic and non-analgesic outcomes after video-assisted thoracoscopic surgery: A propensity matched cohort study
Source: PLoS One. 2021 May 20;16(5):e0252059. doi: 10.1371/journal.pone.0252059 (PMC8136840; doi:10.1371/journal.pone.0252059)
Supplement: S3 Table — (DOCX) [file pone.0252059.s003.docx]

**S3 Table: Subgroup analysis (Based on type of surgery)**

| **Variable** | **Type of surgery** | **GA (n=260)** | **PVB (n=260)** | **p-value** |
| --- | --- | --- | --- | --- |
| Average pain score-24 h | - Non-lung - Minor - Major | - 4.92 (2.64) - 4.79 (2.25) - 4.81 (2.40) | - 5.70 (3.24) - 4.47 (2.61) - 4.98 (2.27) | - 0.850 - 0.218 - 0.429 |
| Average Pain score -24 – 48 h | - Non-lung - Minor - Major | - 4.89 (3.69) - 3.81 (2.82) - 3.86 (2.34) | - 3.88 (3.15) - 3.50 (3.27) - 3.69 (2.42) | - 0.896 - 0.457 - 0.572 |
| Max Pain score - 24h | - Non-lung - Minor - Major | - 8.0 (2.0) - 8.0 (3.0) - 8.0 (3.0) | - 8.0 (2.0) - 8.0 (2.0) - 8.0 (3.0) | - 0.936 - 0.711 - 0.812 |
| Max Pain score - 24 – 48 h | - Non-lung - Minor - Major | - 7.0 (6) - 7.0 (4.0) - 7.0 (3.0) | - 7.0 (4.0) - 6.0 (4.0) - 6.0 (3.0) | - 0.794 - 0.287 - 0.146 |
| MME 24 h | - Non-lung - Minor - Major | - 141.00 (146.00) - 120.00 (105.75) - 126.50 (116.00) | - 63.00 (135.00) - 91.00 (109.00) - 72.50 (81.25) | - **0.011** - **0.001** - **<0.001** |
| MME 24 – 48 h | - Non-lung - Minor - Major | - 56.00 (108.00) - 40.00 (109.00) - 54.00 (89.00) | - 22.00 (63.00) - 32.00 (72.00) - 32.00 (70.75) | - 0.344 - 0.105 - **0.040** |
| LOS | - Non-lung - Minor - Major | - 3.0 (2.0) - 4.0 (4.0) - 4.5 (2.0) | - 4.0 (7.0) - 3.0 (2.0) - 4.0 (2.0) | - 0.572 - **0.003** - **0.033** |
